# Accuracy of MRI in diagnosing intra-articular pathology of the long head of the biceps tendon: results with a large cohort of patients

**DOI:** 10.1186/s12891-019-2654-5

**Published:** 2019-06-01

**Authors:** Jung Youn Kim, Sung-Min Rhee, Yong Girl Rhee

**Affiliations:** 1 Department of Orthopaedic Surgery, Kangnam Sacred Heart Hospital, Hallym University College of Medicine, Seoul, Korea; 2 Shoulder & Elbow Clinic, Department of Orthopaedic Surgery, College of Medicine, Kyung Hee University, Seoul, Korea

**Keywords:** Shoulder, Long head of biceps tendon, Magnetic resonance imaging, Diagnostic efficacy, Diagnostic criteria

## Abstract

**Background:**

It is difficult to diagnose the pathology of the long head of the biceps tendon (LHBT) clinically. This study aimed to determine the diagnostic value of standard non-enhancing magnetic resonance imaging (MRI) for detecting LHBT pathology and identify the most useful diagnostic signs on MRI.

**Methods:**

A total of 554 patients with preoperative 3-Tesla (3 T) MRI who underwent arthroscopic surgery for rotator cuff tears were retrospectively enrolled. Abnormal signs of LHBT on MRI included diameter change, contour irregularity, and alteration of signal intensity. Arthroscopic findings were classified according to tear progress and used as a reference standard: Type I, normal tendon; Type II, hourglass-shaped hypertrophic tendon with fraying extending into the bicipital groove; Type III, partial tear involving less than 50% of tendon width at the intraarticular region without fraying in the bicipital groove; Type IV, partial tear involving more than 50% of tendon width and extending into the bicipital groove; and Type V, complete tear (cutoff) of the tendon. Using receiver operating characteristic, prediction accuracies of MRI findings were assessed compared to those of arthroscopic findings.

**Results:**

Arthroscopic findings showed LHBT pathology in 124 (22.4%) cases. High diagnostic efficacy was achieved when ‘at least 2 abnormal signs’ was set as diagnostic criteria (sensitivity: 77.9%; specificity: 93.7%; positive predictive value: 76.3%). Types II and III lesions showed the highest sensitivities (36.8 and 66.7%, respectively) in abnormal alteration of signal intensity in the parasagittal view while Type IV showed the highest sensitivity (82.3%) in diameter change in axial view. Interobserver agreements were substantial to almost perfect, with kappa value of 0.69–0.81.

**Conclusions:**

The standard non-enhancing 3 T MRI had a high diagnostic value in preoperative detection of LHBT pathology. Its accuracy was increased when diagnostic criterion was set as ‘2 or more abnormal signs (diameter change, contour irregularity, and alteration of signal intensity)’. The single diagnostic sign with the highest sensitivity was alteration of signal intensity in the parasagittal view.

## Background

The long head of the biceps tendon (LHBT) is known to be a relatively frequent cause of anterior shoulder pain [1–3]. Both conservative and surgical treatments can be performed to alleviate symptoms. Treatment method can be selected by taking factors such as comorbid shoulder joint disorders and symptom duration into account [4]. Biceps tenotomy or tenodesis are currently the most popular surgical methods. Both methods are generally recognized for their advantages such as short surgery time, relatively simple techniques, and low costs [5–7]. Speed’s test [8] and Yergason’s test [9] have been developed as physical examinations to detect LHBT pathologies. However, their sensitivities are 32 and 43%, respectively. Their diagnostic performances are considered unsatisfactory [10]. In general, diagnosing LHBT pathology is difficult as it comes concomitantly with other shoulder pathologies such as labral lesions or rotator cuff tears far more frequently than it comes as a solitary lesion [11–13].

Ultrasonography has been traditionally used as an imaging method to diagnose LHBT pathology. It is low in cost, noninvasive, and has an advantage of dynamic study. Its major disadvantages include low diagnostic ability for partial tears and inability to access intraarticular lesion [14, 15]. Magnetic resonance imaging (MRI) is more objective than ultrasonography. It can evaluate intraarticular lesions and partial tears more precisely. Therefore, MRI is preferred as a diagnostic tool for various musculoskeletal pathologies including shoulder joint disorders [11, 16–18]. However, only a small number of known studies have examined MRI’s efficacy for diagnosing LHBT pathology. In addition, they were conducted on small cohorts [17, 19, 20].

In this study, we aimed to determine the diagnostic value of standard non-enhancing magnetic resonance imaging (MRI) for detecting LHBT pathology and identify the most useful diagnostic signs on MRI.

## Methods

### Patient selection

**Fig. 1:**
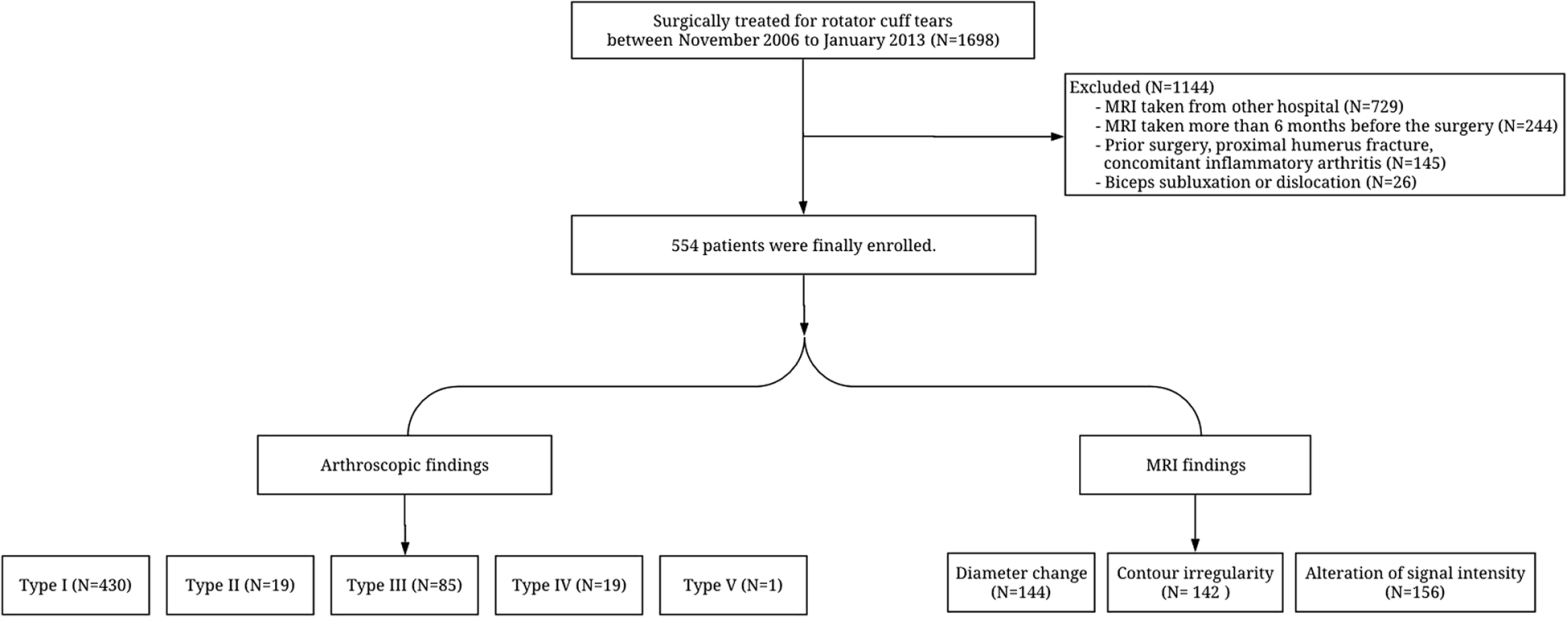
Flow chart depicting included and excluded patients

Medical records of 1698 patients who underwent arthroscopic shoulder surgery for rotator cuff tears between November 2006 and January 2013 were reviewed and prescreened. The inclusion criteria were: the presence of 3-Tesla (3 T) MRI (Achieva; Philips Medical Systems) scans taken in this institution using the same machine within 6 months prior to surgery, LHBT-related arthroscopic findings on operation records, and intraoperative arthroscopic capture images. Of these 1698 patients, 729 whose MRI were taken from other institutions and 244 patients with MRI taken at this institution at more than 6 months before surgery were excluded. Cases (*n* = 145) with prior surgery, proximal humeral fracture, or concomitant inflammatory arthritis were also excluded. As this study focused on the biceps lesion itself, patients with biceps subluxation or dislocation were also excluded (*n* = 26). Thus, 1144 patients were excluded from this study. Finally, 554 patients were enrolled in this study (Fig. 1). As these patients had MRI assessment following our institutional protocol, there was no missing data.

### Arthroscopic findings

**Fig. 2:**
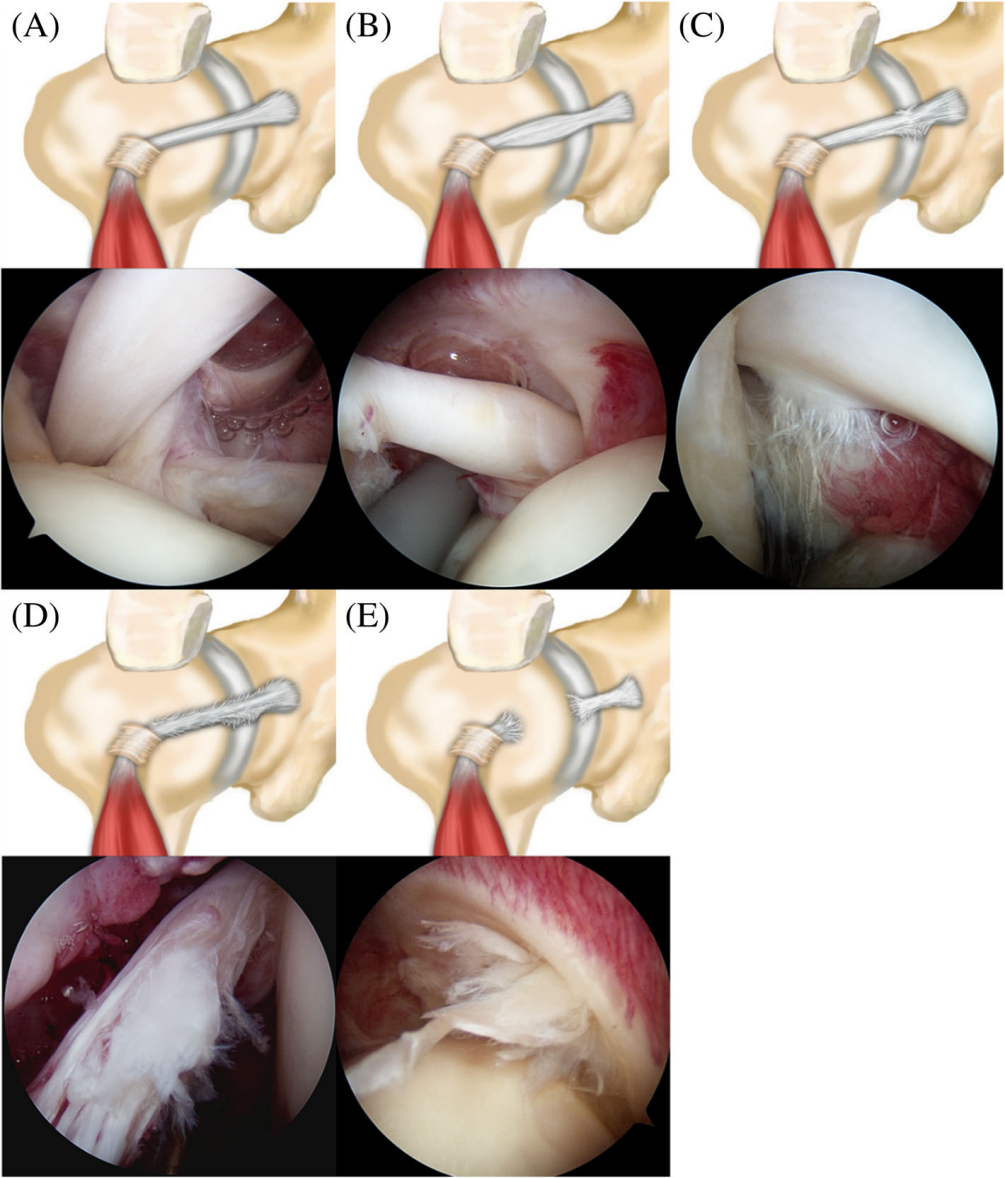
Classification of the long head of the biceps tendon by arthroscopic findings. **a** Type I, Normal tendon. **b** Type II, Hourglass-shaped hypertrophic tendon with extension of fraying into bicipital groove. **c** Type III, Partial tear or fraying involving less than 50% of tendon width at the intraarticular region without fraying in the bicipital groove. **d** Type IV, Partial tear involving more than 50% of tendon width and extending into the bicipital groove. **e** Type V, Complete tear (cutoff) of the tendon

All surgeries were performed by the single senior author. The presence of LHBT pathology was assessed from the intra-articular region into the bicipital groove by pulling the tendon into the joint space using a retriever. Arthroscopic findings on surgical records and intraoperative capture images were reviewed by the senior author who was blinded to the MRI findings. They were used as reference standards for diagnosis of LHBT pathology. To consistently evaluate LHBT pathology, we classified lesions into five types according to the extent of tear: Type I, normal tendon; Type II, hourglass-shaped hypertrophic tendon with fraying extending into the bicipital groove; Type III, partial tear involving less than 50% of tendon width at the intraarticular region without fraying in the bicipital groove; Type IV, partial tear involving more than 50% of tendon width and extending into the bicipital groove; and Type V, complete tear (cutoff) of the tendon (Fig. 2). For those who had high signal intensity in the tendon in MRI finding but with normal thickness and contour, we classified this into Type I. If the continuity of the bicep tendon was intact but showed hyperthrophy after removing frayed tissue by using radiofrequency device, we classified the lesion into Type II. If there was partial loss of continuity, we classified the lesion into Type III or Type IV.

### MRI interpretation

MRI was performed using the 3-T (3 T) (Achieva; Philips Medical Systems) at a single institution. The following sequences were routinely obtained: axial fat-suppressed proton-density-weighted (PDW) (FOV, 140 × 140 mm; TR/TE, 4200/30; flip angle, 90°; matrix, 320 × 240; section thickness, 2.0 mm; and intersection gap, 0.2 mm), axial turbo spin echo (TSE) T2-weighted (FOV, 140 × 140 mm; TR/TE, 3600–4000/80; matrix, 256 × 255; section thickness, 2.0 mm; and intersection gap, 0.2 mm), oblique coronal TSE T1-weighted (FOV, 140 × 140 mm; TR/TE, 500/10; matrix, 320 × 250; section thickness, 2.0 mm; and intersection gap, 0.5 mm), oblique coronal PDW (FOV, 140 × 140 mm; TR/TE, 3500/30; matrix, 320 × 250; section thickness, 2.0 mm; and intersection gap, 0.2 mm), oblique coronal TSE T2-weighted (FOV, 140 × 140 mm; TR/TE, 3500–4000/80; matrix, 350 × 248; section thickness, 2.0 mm; and intersection gap, 0.2 mm), and oblique sagittal TSE T2-weighted (FOV, 140 × 140 mm; TR/TE, 5400–6000/80; matrix, 328 × 240; section thickness, 2.0 mm; and intersection gap, 0.5 mm). In some cases, axial T1-weighted, fat-suppressed T2-weighted coronal or sagittal images were also obtained. As the long head of biceps tendon curves over the head, there could be magic angle phenomenon on T1- and proton-density-weighted sequences using short TE [21]. Therefore, we closely observed and compared T1- and T2-weighted images to reduce magic angle effect. Furthermore, we evaluated for secondary signs of injury such as tendinopathy or effusion around the tendon to minimize false-positive.

**Fig. 3:**
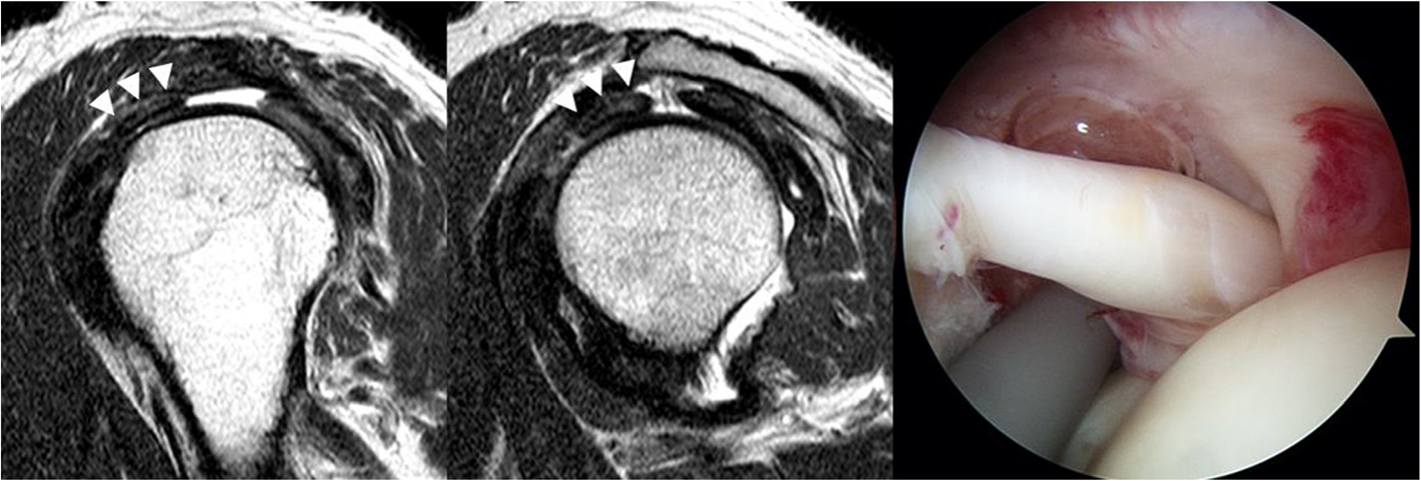
Two consecutive T2-weighted parasagittal MRI showing abrupt diameter change and high signal intensity inside of the tendon (white arrowhead). The contour of the tendon was smooth and uniform. This patient’s MRI revealed hypertrophy in the long head of the biceps tendon (Type II)

**Fig. 4:**
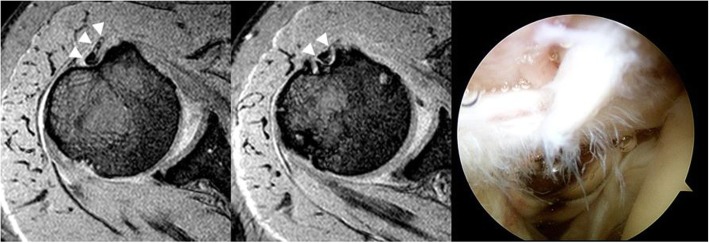
A T2-weighted axial MRI showing severe contour irregularity of the tendon at the bicipital groove (white arrowhead). This patient’s MRI revealed a complex tear involving nearly the full width of the long head of the biceps tendon in the bicipital groove (Type V)

**Fig. 5:**
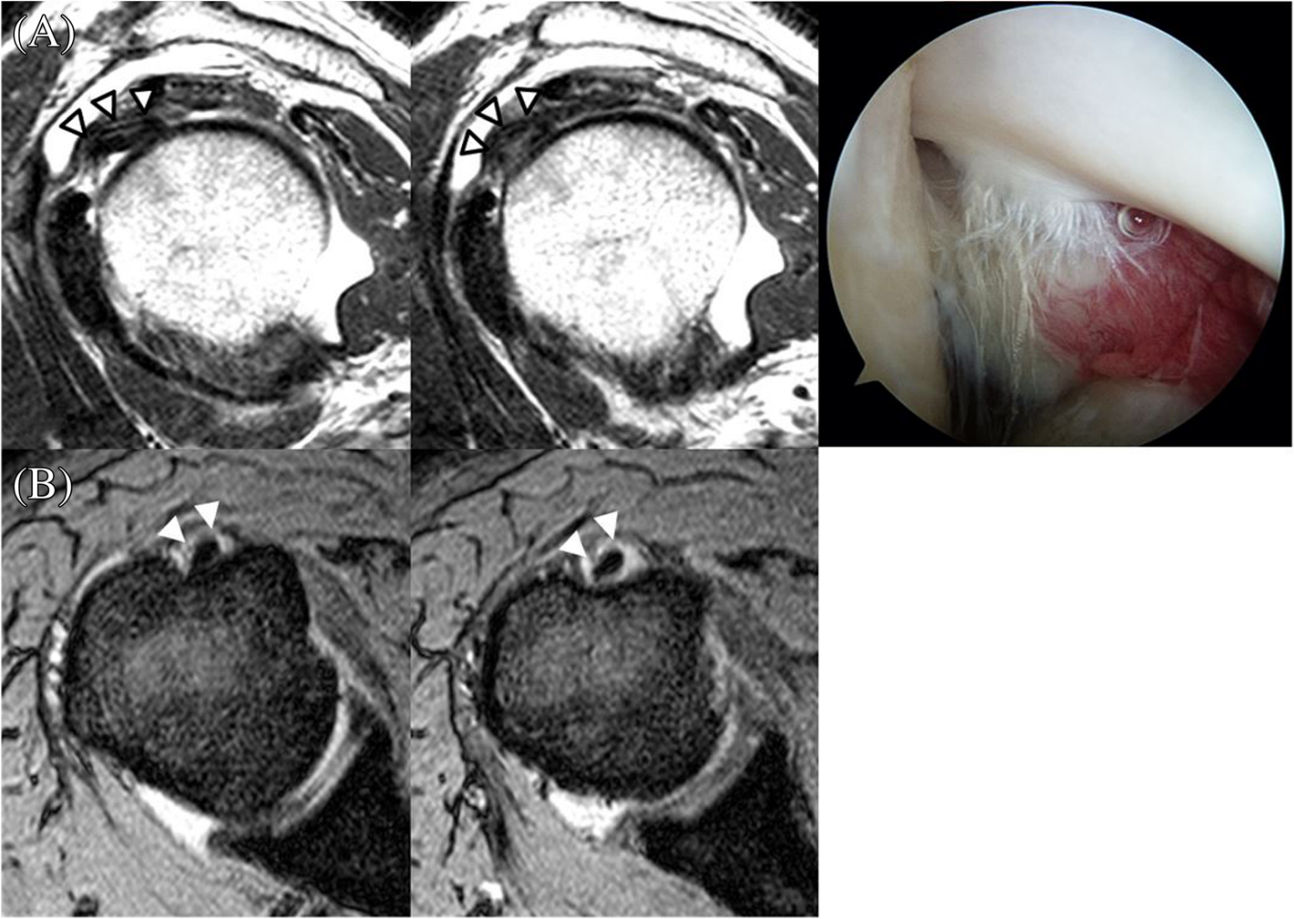
**a** Two consecutive T2-weighted parasagittal MRI showing mild contour irregularity and heterogenous high signal intensity inside the tendon (white arrowhead). **b** Two consecutive T2-weighted axial MRI of the same patient showed high signal intensity surrounding the tendon, suggesting effusion in bicipital groove (Type III)

All images were reviewed independently by two fellowship-trained orthopedic surgeons specializing in shoulder joint. They were also trained in shoulder MRI interpretation by co-working with musculoskeletal radiologists. Every interpretations of MRIs including mismatches was re-assessed by the senior radiologist. If there was discordance of MRI findings between shoulder surgeons and the senior radiologist, the final interpretation followed the interpretation of the senior radiologist. They were not aware of arthroscopic finding, the reference standard. After finishing all interpretations, interobserver agreement between the two observers was assessed. Data were then pooled from the two observer’s interpretations. LHBT was assessed for abnormal findings in axial and parasagittal planes. Signs used for interpretation included: (1) diameter change, (2) contour irregularity, and (3) alteration of signal intensity (Figs. 3, 4 and 5). Each sign was categorized as normal or abnormal.

### Statistical analysis for diagnostic accuracy

Sensitivity, specificity, positive predictive value (PPV), negative predictive value (NPV), and accuracy were calculated to assess diagnostic efficacy for pooled LHBT abnormalities. PPV was defined as the probability of disease given a positive test result while NPV was defined as the probability of the absence of disease given a negative test result. The same factors were calculated again for each LHBT lesion type. These values were then separately calculated by varying the decision criteria for LHBT pathology from at least one abnormal sign to three abnormal signs. Interobserver agreement was assessed using weighted Cohen’s kappa (*k*: ≤ 0.00, poor; 0.01–0.20, slight; 0.21–0.40, fair; 0.41–0.60, moderate; 0.61–0.80, substantial; 0.81–1.00, almost perfect) [22].

In order to determine the optimal number (cutoff value) of abnormal signs to diagnose an LHBT pathology, receiver operating characteristic (ROC) curve and Youden index were used [23]. The ROC curve was generated by plotting our sample values’ sensitivity (true positive rate) along the Y-axis and specificity (false positive rate) on the X-axis. The accuracy of the test depended on how well the test separated the group tested into those with and without the disease in question. Accuracy was measured by the area under the ROC curve. An area of 1 represented a perfect test while an area of 0.5 represented a worthless test. Youden index used the maximum of vertical distance of ROC curve from the point (x,y) on diagonal line (chance line). By maximizing sensitivity + specificity across various cut-off points, the optimal number (cutoff value) was calculated [23].

## Results

### Overall results

Reviewing arthroscopic findings from operation records identified 124 (22.4%) cases of pathologic LHBT and 430 (77.6%) cases of normal LHBT (Type I). Of 124 abnormal LHBT cases, 19, 85, 19, and 1 were type II, type III, type IV, and type V lesions, respectively (Table 1). In the MRI, 326 cases showed normal LHBT, while 228 cases showed abnormal appearance (Table 2). Among 554 patients, 16 patients who had normal appearance on MRI showed abnormal arthroscopic finding.

**Table 1:** Incidence of Long Head of Biceps Tendon Types by Arthroscopic Findings

	Incidence (%)
Type I (normal)	47.6% (430/554)
Type II (hypertrophic)	3.4% (19/554)
Type III (partial tear < 50%)	15.4% (85/554)
Type IV (partial tear > 50%)	3.4% (19/554)
Type V (complete)	0.2% (1/554)

**Table 2:** The Arthroscopic Findings and the MRI Findings of the Long Head of Biceps Tendon

Arthroscopic findings	Type I	Type II	Type III	Type IV	Type V
MRI findings					
Normal	310	6	10	0	0
Diameter change	26	0	4	0	0
Contour irregularity	33	2	2	0	0
Alteration of signal intensity	33	3	5	0	0
Two abnormal findings	22	4	19	2	0
Three abnormal findings	6	4	45	17	1
Total	430	19	85	19	1

Interobserver agreements between the two observers were substantial to almost perfect, with kappa values ranging from 0.69 to 0.81. (Table 3) Therefore, diagnostic efficacy was calculated using data pooled from the two observers.

**Table 3:** Diagnostic Efficacy of MRI in Pathology of the Long Head of the Biceps Tendon

(%)	Diameter change		Contour irregularity		Alteration of signal intensity	
	Parasagittal	Axial	Parasagittal	Axial	Parasagittal	Axial
Sensitivity	52.0	66.7	56.0	58.2	66.6	59.7
Specificity	93.6	94.4	94.3	92.9	91.8	93.3
Accuracy	83.0	88.3	85.0	85.0	86.1	85.6
PPV^a^	69.2	78.6	72.6	70.9	71.2	71.7
NPV^a^	84.7	91.0	86.9	88.2	90.2	87.5
Cohen’s kappa	0.69	0.71	0.74	0.78	0.81	0.79

^a^*PPV* positive predictive value, *NPV* negative predictive value

### Diagnostic efficacy by pooled interpretation

Table 3 shows diagnostic efficacy of non-enhancing standard MRI by pooled interpretation from the two observers. Results showed moderately high sensitivities, very high specificities, and very high accuracies in differentiating normal tendons from pathologic tendons. Diameter changes in the axial view and alteration of signal intensity in the parasagittal view showed higher sensitivity than other items (66.7 and 66.6% with kappa value of 0.71 and 0.81, respectively). Specificity was very high (≥ 90%) for all items.

### Optimal number of diagnostic signs to detect LHBT lesion

Defining the diagnostic criterion for pathologic LHBT as ‘at least one abnormal sign’ among six interpretation signs resulted in a high sensitivity (85.6%) but a relatively low PPV (49.2%). Defining the diagnostic criterion as ‘at least 2 abnormal signs’ resulted in higher specificity (93.7%) and moderately high PPV (76.3%) whereas sensitivity (77.9%) decreased slightly compared to the diagnostic criterion of ‘at least 1 abnormal sign’. Defining the diagnostic criterion as ‘three abnormal signs’ yielded the highest specificity (96.3%) and PPV (85.8%) whereas sensitivity decreased substantially (66.9%) (Table 4).

**Table 4:** Diagnostic Efficacy According to Various Decision Criteria

(%)	1 abnormal sign	2 abnormal signs	3 abnormal signs
Sensitivity	85.6	77.9	66.9
Specificity	73.6	93.7	96.3
Accuracy	76.6	89.2	89.5
PPV^a^	49.2	76.3	85.8
NPV^a^	94.5	93.1	90.1

^a^*PPV* positive predictive value, *NPV* negative predictive value

**Fig. 6:**
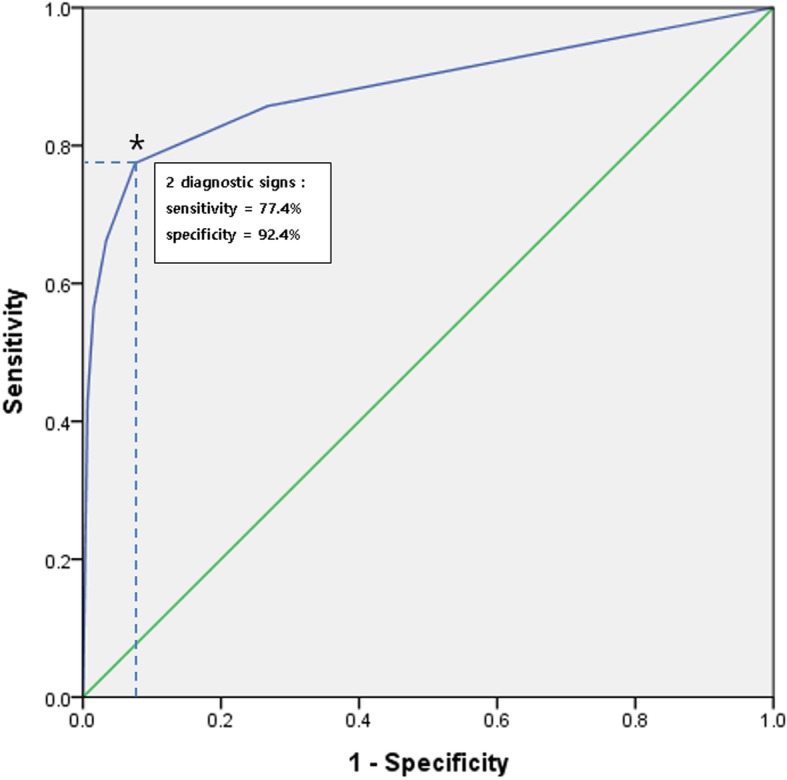
Receiver operating characteristic (ROC) curve illustrating the ability of MRI diagnostic signs to discriminate pathologic long head of the biceps tendon (LHBT) cases from normal LHBT. The asterisk indicates the optimal point (two diagnostic signs) which balances the sensitivity and specificity (77.4 and 92.4%, respectively) of the test. The ROC curve has an area under the curve of 0.883 (95% confidence interval: 0.842–0.911)

ROC curves were used to determine the optimal number of diagnostic signs that was the most effective for diagnosing pathologic LHBT (Fig. 6). The area under the ROC curve was 0.883 and the accuracy of diagnostic method in this study was proved to be very good. The point where the slope of the ROC curve abruptly changed the closest from the upper left corner of the graph was the optimal point in which the sensitivity and specificity were balanced (Fig. 6). From that point, it was found that the optimal number of diagnostic signs was 2 in which the sensitivity and specificity were well balanced, with values of 77.4 and 92.4%, respectively.

Using ROC curves, the optimal cutoff value capable of differentiating Type IV lesion from other types was determined. The area under the ROC curve was 0.887. By defining the criterion as three or more diagnostic signs, differentiation could be achieved with sensitivity of 80.6% and specificity of 91.2%.

### Diagnostic efficacy according to LHBT tear type

Diagnostic efficacy of MRI for each LHBT tear type (II-IV) was analyzed. Type V was excluded from this analysis because there was only one case. The sensitivity increased as lesion type became more severe (from 68.4 to 100.0%). The lesion type that was the least accurately diagnosed with MRI interpretation was type II, with lowest sensitivity among groups (68.4% by one sign and 57.9% by two signs). Defining the diagnostic criterion for LHBT abnormality as ‘at least two abnormal signs’ resulted in slightly lower sensitivity compared to the criterion of ‘one abnormal sign’. However, very high values were obtained for other diagnostic variables (specificity, accuracy, PPV, and NPV) (Table 5).

**Table 5:** Diagnostic Efficacy According to Lesion Types

(%)	Type I	Type II		Type III		Type IV	
		1 sign	2 signs	1 sign	2 signs	1 sign	2 signs
Sensitivity	72.8	68.4%	57.9%	83.7%	74.4%	100.0%	100.0%
Specificity	85.2	72.8%	92.0%	72.8%	92.0%	72.8%	92.0%
Accuracy	75.6	72.6%	90.5%	73.8%	90.4%	74.3%	92.5%
PPV^a^	94.2	10.1%	24.4%	23.7%	48.5%	18.3%	43.3%
NPV^a^	48.4	98.1%	98.0%	97.8%	97.3%	100.0%	100.0%

^a^*PPV* positive predictive value, *NPV* negative predictive value

### Most sensitive diagnostic sign according to LHBT tear type

MRI signs and planes that most sensitively detected the abnormality of each LHBT lesion type were analyzed (Table 6). For type II intra-articular lesion, the parasagittal plane was the most efficient plane to detect the lesion. Its combination with alteration of signal intensity showed the highest sensitivity. However, type II was difficult to detect by MRI, with sensitivity of only 36.8%. For type III, the highest sensitivity was 66.7% when alteration of signal intensity in the parasagittal plane was used as with type II. For Types IV and V, the highest sensitivity was shown when diameter change in the axial plane and alteration of signal intensity in the parasagittal plane were used (type IV: 71.7 and 58.7%; type V: 92.3 and 100.0%, respectively). Among all MRI signs, contour irregularity was found to be the least accurate diagnostic sign with the lowest sensitivity for all lesion types.

**Table 6:** The Most Reliable Diagnostic Sign According to the Lesion Type

Lesion Type	Diagnostic sign	^a^MRI plane	Sensitivity (%)
Type II	Alternation of signal intensity	Parasagittal	36.8
Type III	Alternation of signal intensity	Parasagittal	66.7
Type IV	Diameter change	Axial	82.3
	Alteration of signal intensity	Parasagittal	76.5

^a^*MRI* Magnetic resonance imaging

## Discussion

LHBT lesions are rarely present as solitary lesions. They are often concomitant with rotator cuff tears or labral lesions. Skendzelet al. [14] have reported that 96.2% of LHBT tear cases are comorbid with supraspinatus tendon tears based on MRA findings. Murthiet al. [2] have performed histologic analysis of specimens obtained via tenosynovectomy of LHBT on 200 patients who underwent arthroscopic subacromial decompression for impingement syndrome and reported that normal biceps tendon cases account for only 18% of total cases while 76% of all cases have chronic inflammation or fibrosis. The incidence of biceps pathology is increased in proportion with the extent of rotator cuff tear. Accordingly, abnormalities in biceps tendons can be major causes of shoulder pain with other shoulder lesions. In the present study, MRI findings showed LHBT pathology in 124 (22.4%) cases, different from the incidence reported by in the previous study [2]. Such difference in incidence of biceps pathology might be due to difference in histological approach or difference in the definition of normal biceps tendon.

It can be a great challenge to identify the pathology of shoulder pain. Because LHBT pathology is highly likely to be comorbid with other shoulder pathologies, physical examinations often show nonspecific results or LHBT is overlooked in deference to another major pathology. Therefore, it is important to detect the presence of LHBT lesion and be prepared before setting up a treatment plan [2, 3]. Various noninvasive imaging methods have been employed to determine the underlying pathology of shoulder pain and help choose treatment method. Ultrasonography and MRI are broadly and actively applied in orthopedic surgery fields. Diagnostic arthroscopy is generally the last choice because it is an invasive procedure.

As a preoperative diagnostic tool for LHBT pathology, ultrasonography is rapid and cost-effective. It can be conducted in the outpatient department. Skendzel et al. [14] have analyzed the diagnostic accuracy of preoperative sonography performed on 66 patients and revealed its high diagnostic accuracies for complete tear of the biceps tendon, with sensitivity of 88% and specificity of 98%. However, sonography is inadequate for diagnosing partial tears (sensitivity, 27%; specificity, 100%).

Studies on MRI-assisted diagnosis of LHBT pathology have emerged recently with inconsistent results [11, 17, 19, 20, 24]. Zanetti et al. [20] have reported that MRA has adequately high sensitivity for distinction. In their study, two independent observers compared images obtained from 42 patients with their arthroscopic findings to determine the degree of agreement between MRI and arthroscopic findings (observer 1: sensitivity of 92% and specificity of 56%; observer 2: sensitivity of 89% and specificity of 81%). However, the interobserver agreement was low (kappa value: 0.39).

In the present study, both observers who compared interpretations of standard non-enhancing MRI and arthroscopic findings showed high sensitivity and specificity. Interobserver agreements were substantial to almost perfect, with kappa values ranging from 0.68 to 0.81. It was found that diameter change on the axial plane and alteration of signal intensity on the parasagittal plane accurately differentiated LHBT pathologies at a high rate (accuracy ≥80%). Setting the diagnostic criterion as ‘at least two positive signs’ to detect LHBT lesion yielded the most balanced results for all parameters. This suggests that a comprehensive interpretation of various signs in different planes rather than basing diagnosis on one definitive sign can enhance diagnostic accuracy. This makes sense as the biceps tendon runs in the medial-to-lateral direction in the intraarticular region and superior-to-inferior direction when it passes into the bicipital groove.

Diagnostic accuracy was increased when lesion severity was increased. Type II lesion showed the lowest sensitivity. Thus, MRI was considered inadequate for diagnosing this type of lesion. On the other hand, more severe lesions such as type IV and type V lesion that extend into the bicipital groove were more accurately diagnosed. Because tenodesis or tenotomy should be often considered in this type of lesion, it is required to ensure accurate diagnosis prior to surgery. Our analysis showed a high probability for the presence of type III or type IV lesion with diagnostic criterion set as ‘three abnormal signs’. Interpreting MRIs based on these diagnostic criteria can be useful for surgeons to set up a preoperative plan and educate the patient.

The 3 T MR machine was first introduced in 1999. Its advantages over conventional machines with low magnetic field strengths include a higher signal-to-noise ratio, improved spatial resolution, and shorter acquisition time [25]. Many previous studies that reported low diagnostic efficacy of MRI for detection of LHBT lesions [11, 20, 24] used 1.0 T or 1.5 T machines which might make image interpretation difficult. The high diagnostic efficacy obtained in this study may be attributed to the use of 3 T machines. Strengths of this study were: 1) we confirmed that standard non-enhancing MRI had sufficient diagnostic efficacy to detect biceps pathology if adequate criteria were used; 2) we presented a novel classification method according to tear progression and demonstrated that MRI sign and plane were the most suitable for diagnosing each classification type; 3) this study was carried out on a much larger group of patients than previous studies. All these advantages are sufficient to prove the diagnostic value of MRI.

However, this study also had some limitations. First, there was only one type V lesion. Thus, statistically significant value could not be obtained. Because type V (complete biceps tendon tear) has already been identified with high diagnostic accuracy in previous studies by both MRI and ultrasonography, we excluded type V from our analysis so that it had little influence on overall results of our study. Second, we described MRI interpretations only as normal or abnormal without differentiating lesion types. As a result, the diagnostic power for lesion type differentiation could not be determined. Third, the lack of evaluation of “hidden lesion” of extra-articular LHBT in arthroscopy is a limitation of this investigation [26]. Fourth, this study focused on the lesion of biceps tendon itself and thus, we did not include dislocations or subluxations from the bicipital groove as a lesion. Fifth, since this study was conducted in a population undergoing surgery for rotator cuff disease, the prevalence of LHBT pathology probably would be higher than in other populations which affects the positive predictive value and negative predictive value. Lastly, as this study was on the diagnostic accuracy of MRI in diagnosing intra-articular pathology of the long head of the biceps tendon, we did not assess the correlation with clinical signs of biceps pathology. Further studies are needed to address these limitations.

## Conclusion

In conclusion, standard non-enhancing MRI using a 3 T MRI machine has highly reliable diagnostic value for preoperative detection of LHBT pathology. Diagnostic accuracy is increased as lesion severity is increased. Among changes in diameter, contour irregularity, and alteration of signal intensity, reading with two or more diagnostic signs showed the highest accuracy. The single diagnostic sign that was the most sensitive for accurate diagnosis was the alteration of signal intensity in the parasagittal view.

## Data Availability

The datasets used and/or analyzed during the current study are available from the corresponding author on reasonable request.

## Abbreviations

3 T: 3-Tesla
LHBT: Long head of the biceps tendon
MRA: Magnetic resonance arthrography
MRI: Magnetic resonance imaging
NPV: Negative predictive value
PPV: Positive predictive value
ROC: Receiver operating characteristic
